# Effects of periodontal initial therapy combined with orthodontic treatment on anterior tooth function and inflammatory factors in gingival crevicular fluid in patients with periodontal disease induced anterior tooth displacement

**DOI:** 10.12669/pjms.39.6.7135

**Published:** 2023

**Authors:** Yan Liu, Xie Shi, Gengbing Lin, Nan Guo

**Affiliations:** 1 Yan Liu, Department of Stomatology, Fujian Provincial People’s Hospital, Affiliated People’s Hospital of Fujian University of Traditional Chinese Medicine, Fuzhou 350005, Fujian, China; 2 Xie Shi, Department of Orthodontics, Fujian Key Laboratory of Oral Diseases & Fujian Provincial Engineering Research Center of Oral Biomaterial & Stomatological Key lab of Fujian College and University, School and Hospital of Stomatology, Fujian Medical University & Institute of Stomatology, Fujian Medical University, Fuzhou 350002, Fujian, China; 3 Gengbing Lin, Department of Stomatology, Fujian Provincial People’s Hospital, Affiliated People’s Hospital of Fujian University of Traditional Chinese Medicine, Fuzhou 350005, Fujian, China; 4 Nan Guo, Department of Stomatology, Fujian Provincial People’s Hospital, Affiliated People’s Hospital of Fujian University of Traditional Chinese Medicine, Fuzhou 350005, Fujian, China

**Keywords:** Periodontal initial therapy, Orthodontics, Periodontal disease, Anterior tooth displacement

## Abstract

**Objective:**

To evaluate the effects of periodontal initial therapy combined with orthodontic treatment on anterior tooth function and inflammatory factors in gingival crevicular fluid in patients with periodontal disease induced anterior tooth displacement.

**Methods:**

This was a clinical comparative study. A total of 140 patients with anterior teeth displacement caused by periodontal disease in Fujian University of Traditional Chinese Medicine from May 2020 to May 2022 were selected and randomly divided into two groups. Patients in the control group received periodontal initial therapy, and those in the study group were provided with orthodontic treatment on the basis of initial therapy. Further comparative analysis was performed focusing on the clinical effects of the two groups, the changes of probing depth, anterior overjet degree, oral function and inflammatory factors in gingival crevicular fluid before and after treatment.

**Results:**

The efficacy of the study group was significantly higher than that of the control group(p=0.00). After treatment, the probing depth, the anterior overjet degree and the rate of bleeding on probing in the study group were significantly lower than those in the control group(P=0.00). Furthermore, the proportion of tooth mobility degrees I, II and III in the study group was significantly lower than that in the control group after treatment(P<0.05). The levels of post-treatment inflammatory factors in the study group were significantly lower than those in the control group(p=0.00).

**Conclusion:**

Periodontal initial therapy combined with orthodontic treatment has a significant effect on anterior teeth displacement caused by periodontal disease, which deserves promotion clinically.

## INTRODUCTION

It has a serious impact on the gingiva and periodontal tissue of patients. Periodontitis is the most common periodontal disease, and it has been confirmed by multiple clinical studies that its onset is related to bacterial infection.^1^,^2^ It is a chronic inflammatory oral disease in which pathogenic bacteria invade periodontal supporting structure and cause periodontal tissue destruction. The destruction of periodontal tissue is mainly caused by excessive host response to produce cytokines, such as interleukin-6(IL-6) and tumor necrosis factor (TNF)-α.^3^,^4^ It plays an extremely important role in chronic inflammatory reactions such as periodontitis; and it can act on immune cells to induce tissue destruction and bone absorption.^5^

Periodontal initial therapy has been considered as an important therapeutic option for periodontitis and is widely used clinically. As suggested by Manresa et al.^6^, after the completion of periodontal initial therapy and the cessation of inflammation, further supportive periodontal treatment should be adopted to reduce the possibility of reinfection and disease progression. At present, there is a continuous development of orthodontic treatment technology, which provides a new direction for the treatment of anterior tooth displacement caused by periodontal disease. Our objective was to evaluate the effects of periodontal initial therapy combined with orthodontic treatment which was applied to treat patients with anterior tooth displacement caused by periodontal disease.

## METHODS

This was a clinical comparative study. A total of 140 patients with anterior teeth displacement caused by periodontal disease who were admitted to Fujian University of Traditional Chinese Medicine from May 2020 to May 2022 were selected and randomly divided into two groups, with 70 cases in each group. There was no significant difference in general data between the two groups, suggesting the comparability between groups (Table-I).

**Table-I:** Comparison of general data between the study group and the control group (*χ̅*±*S*).

Indexes	Study group(n=70)	Control group(n=70)	t/χ^2^	P
Age(years)	41.50±8.26	42.02±8.31	0.37	0.71
Male(n %)	34(48.6%)	42(60%)	1.84	0.18
Course of disease(d)	5.73±1.68	5.58±1.43	0.59	0.57
BMI(kg/m^2^)	25.36±4.32	25.82±4.30	0.64	0.53
***Complication***				
Dental caries	42(60%)	40(57%)	0.12	0.73
Pulpitis	15(21.4%)	17(24.3%)	0.16	0.69
Dental trauma	9(12.9%)	8(11.4%)	0.06	0.80
Opsigenes	4(6%)	5(7%)	0.12	0.73
***Bad habits***				
Smoking history	36(51.4%)	38(54.3%)	0.11	0.74
Fond of uncooked and cold food	22(31.4%)	30(42.9%)	1.96	0.16
High-fat diet	26(37.1%)	21(30%)	0.81	0.37
Previous treatment				
Root canal therapy	20(28.6%)	23(32.9%)	0.30	0.58
Repair of tooth defect	32(45.7%)	34(48.6%)	0.11	0.74

P>0.05.

### Ethical Approval

The study was approved by the Institutional Ethics Committee of Affiliated People’s Hospital of Fujian University of Traditional Chinese Medicine (No: 202287; Date: December 09, 2021), and written informed consent was obtained from all participants.

### Inclusion criteria:

- Patients who met the diagnostic criteria for periodontal disease^7^;
- Patients with displacement of anterior teeth, protrusion of anterior teeth, widening of interdental space or loose teeth;
- Patients who were sensitive to initial treatment of periodontal disease and showed improvement after treatment;
- Patients with complete medical history and high compliance;
- Patients who agreed to the study protocol and signed the consent form by themselves or their families.

### Exclusion criteria:

- Patients with cognitive dysfunction or mental diseases;
- Patients with poor oral hygiene habits;
- Patients with hyperthyroidism, diabetes, tuberculosis, coagulation dysfunction, etc.;
- Patients with severe heart, liver and kidney dysfunction;
- Patients with severe malnutrition.

Patients in the control group received periodontal initial therapy, including oral hygiene, supragingival scaling and subgingival scaling. Meanwhile, the residual calculus in the periodontal pocket was removed postoperatively to make the gums and tooth surfaces fit. Patients in the study group were provided continuously with orthodontic treatment on the basis of the treatment in the control group. The specific method was to implement orthodontic treatment when the periodontal symptoms were significantly improved and stabilized. The edgewise appliance was used for correction, with the adjustment of the orthodontic force properly to keep an appropriate strength. The orthodontic treatment lasted generally 6-12 months. After orthodontic treatment, patients were instructed to wear bite guard to reduce the recurrence of anterior tooth displacement.

### Outcome measures:

### Clinical effects

Significant effective: restoration of the anterior teeth to a beautiful appearance, normal masticatory function and the tooth mobility degree of below Grade-I, and disappearance of periodontitis symptoms. Effective: restoration of the anterior teeth to a beautiful appearance, the tooth mobility degree of Grade-I~II, and disappearance of periodontitis symptoms to some extent. Ineffective: no improvement of the anterior tooth displacement, weak teeth, the tooth mobility degree of Grade-I~II, and obvious periodontitis symptoms. The effective rate=(cases of significant effective+effective)/100.

### Periodontal tissue recovery

Including probing depth, anterior overjet degree, alveolar bone height, and rate of bleeding on probing.

The tooth mobility degree was observed.Before treatment and 12 months after treatment, dental function was compared by the Behavioral Assessment Scale of Oral Functions prepared by Stratton.^8^ A higher score might indicate a better functional status.

Inflammatory factors in gingival crevicular fluid: The levels of inflammatory factors such as TNF-α and IL-6 were detected by enzyme-linked immunosorbent assay(ELISA) in the venous blood samples collected from the enrolled patients.

### Statistical analysis

All data of this study were statistically analyzed with SPSS 20.0 software, and the measurement data were expressed in (*χ̅*±*S*). Two independent sample t-test was used for inter-group data analysis, paired t-test for intra-group data analysis, and c^2^ test for rate comparison. The confidence interval is 95%. P<0.05 was considered to indicate a statistically significant difference.

## RESULTS

The efficacy of the study group was significantly higher than that of the control group(p=0.00) (Table-II). After treatment, the probing depth, the anterior overjet degree and the rate of bleeding on probing in the study group were significantly lower than those in the control group(P=0.00) (Table-III). The proportion of tooth mobility degrees I, II and III in the study group was significantly lower than that in the control group after treatment (degrees I and II, p=0.00; degree III, p=0.02) (Table-IV).

**Table-II:** Comparative analysis of clinical effect between the two groups (*χ̅*±*S*).

Groups	Significant effective	Effective	Ineffective	Effective rate
Study group (n=70 )	61	4	5	65(92.8%)
Control group (n=70 )	10	24	36	34(48.5%)
c^2^				33.41
P				0.00

p<0.05.

**Table-III:** Comparative analysis of periodontal tissue recovery between the two groups(*χ̅*±*S*).

Outcome measures		Study group(n=70)	Control group(n=70)	t/χ^2^	p
Probing depth(mm)	Before treatment	5.73±1.20	5.68±1.13	0.25	0.80
	After treatment*	3.78±1.04	4.63±1.25	4.37	0.00
Anterior overjet degree(mm)	Before treatment	5.27±2.06	5.18±2.03	0.26	0.78
	After treatment*	2.36±1.87	4.03±2.34	4.66	0.00
Alveolar bone height(mm)	Before treatment	5.17±0.46	5.13±0.38	0.56	0.58
	After treatment*	5.03±0.44	5.12±0.28	1.44	0.15
Rate of bleeding on probing (%)	Before treatment	87%	85%	0.16	0.68
	After treatment*	7%	21%	8.14	0.00

P>0.05.

**Table-IV:** Comparative analysis of tooth mobility between the two groups before and after treatment (*χ̅*±*S*).

Outcome measures		Study group(n=70)	Control group(n=70)	t/c^2^	p
Degree I	Before treatment	16(22.8%)	18(25.7%)	0.24	0.62
	After treatment*	0	11(15.7%)	11.64	0.00
Degree II	Before treatment	41(58.5%)	40(57.1%)	0.08	0.77
	After treatment*	2(2.9%)	16(22.9%)	17.68	0.00
Degree III	Before treatment	13(18.7%)	12(17.2%)	0.14	0.71
	After treatment*	3(4.3%)	9(12.9%)	5.21	0.02

* p<0.05.

After treatment, the masticatory function, fixation, aesthetics and comfort of the study group were significantly better than those of the control group(P=0.00) (Table-V). After treatment, TNF-α and IL-6 in both groups were lower than those before treatment(p=0.00). Besides, the post-treatment levels of TNF-α and IL-6 in the study group were significantly lower than those in the control group(p=0.00) (Table-VI).

**Table-V:** Comparative analysis of oral function between the two groups before and after treatment (*χ̅*±*S*).

Outcome measures		Study group(n=70)	Control group(n=70)	t	p
Masticatory function	Before treatment	2.62±0.36	2.73±0.41	1.68	0.09
	After treatment*	4.33±0.52	3.47±0.53	9.69	0.00
Fixation	Before treatment	2.31±0.50	2.34±0.28	0.44	0.65
	After treatment*	3.12±0.48	2.63±0.62	5.23	0.00
Aesthetics	Before treatment	2.85±0.46	2.79±0.42	0.81	0.42
	After treatment*	4.43±0.32	3.85±0.77	5.82	0.00
Comfort	Before treatment	2.63±0.51	2.60±0.46	0.37	0.72
	After treatment*	3.75±0.68	3.18±0.53	5.53	0.00

p>0.05.

**Table-VI:** Comparative analysis of changes in inflammatory factors between the two groups before and after treatment (*χ̅*±*S*).

Indicators		Study group*(n=70)	Control group*(n=70)	t	p
TNF-α(ng/L)	Before treatment	28.03±6.33	27.82±7.02	0.58	0.42
	After treatment*	14.36±3.71	23.48±3.63	15.10	0.00
		t	17.46	14.28	
		p	0.00	0.00	
IL-6(ng/L)	Before treatment	11.56±2.37	11.35±2.54	0.38	0.62
	After treatment*	6.73±1.75	8.81±2.28	3.74	0.00
		t	14.37	11.60	
		p	0.00	0.00	

* p<0.05.

## DISCUSSION

In our study, the efficacy of periodontal initial therapy combined with orthodontic treatment was significantly higher than that of periodontal initial therapy alone, suggesting a significant effect of orthodontic treatment on anterior teeth displacement. And the probing depth, the anterior overjet degree and the rate of bleeding on probing of patients in the study group were significantly lower than those in the control group. It supports a reduced potential risk of traumatic occlusion and improved periodontal condition of the anterior teeth after restoration of the anterior teeth with orthodontic treatment. Generally, orthodontic treatment is based on the slow reconstruction of alveolar bone around the root. Therefore, it is important to follow the correct biological force during orthodontic treatment to strengthen the teeth and reduce the degree of root absorption.^9^

In our study, there was an improvement in tooth mobility to certain extent in patients. Our study indicated that the proportion of tooth mobility degrees I, II and III in the study group was significantly lower than that in the control group after treatment. Simultaneously, the post-treatment masticatory function, fixation, aesthetics and comfort of the study group were evidently better than those of the control group. All these findings suggest that there will be obvious changes of periodontal tissue and alveolar bone after orthodontic treatment than that before treatment, which creates beneficial conditions for periodontal tissue regeneration.

Periodontal disease is an oral disease that has a high incidence clinically. Periodontitis is the most common form that accounts for >95% of the total number of patients with periodontal disease.^10^ Its specific pathogenesis is related to immune system disorders and microbial infections. The main clinical symptoms of periodontitis are gingival inflammation, gingival bleeding, periodontal pocket formation, alveolar bone absorption, tooth loosening, etc. Tooth displacement can be induced by periodontal tissue destruction, bite force change, etc. Anterior tooth displacement is common in clinical practice, and in serious cases, it even shifts and falls off, which seriously affects the patients’ normal life and interferes with the appearance.^11^

Periodontal initial therapy has been regarded to be the basic treatment of periodontitis.^12^ Periodontal initial therapy can effectively remove subgingival plaque calculus and diseased cementum, so as to keep the root surface smooth and prevent plaque reattachment.^13^ But it cannot fundamentally improve the oral condition of patients, especially those with loose teeth and displaced anterior teeth. Orthodontic treatment offers another feasible therapeutic option for treating the symptoms of anterior teeth displacement and tooth loosening caused by periodontal disease. It has been documented that after orthodontic treatment, periodontal tissue and alveolar bone showed obvious changes compared with that before treatment, creating favorable conditions for periodontal tissue regeneration.^14^

According to previous research,^15^,^16^ periodontal infection has a mutual relationship with systemic diseases. Prior study has detected the significantly increased levels of IL-6, IL-8 and TNF-α in the serum and gingival crevicular fluid of patients with periodontitis.^17^,^18^, ^19^ In our study, the levels of serum inflammatory factors IL-6 and TNF-α in the enrolled patients were improved after 12 months of treatment compared with those before treatment, with more significant decrease in the study group than those in the control group. As reported by Zasciurinskienė et al.^20^ orthodontic treatment has a significant effect on the periodontal status of periodontitis patients with dental plaque, and can obviously improve the probing depth. Verrusio et al.^21^ also discovered in their research that periodontal indices increased after orthodontic treatment, accompanied by the accumulation and composition of subgingival microbiota, thus reducing inflammation and enhancing tooth firmness. Collectively, it is speculated that through orthodontic treatment, the impact of traumatic occlusion can be eliminated, and the gingival inflammation can be alleviated.

### Limitations

It includes the smaller sample size and relatively shorter period of follow-up. Our future research will be continued based on the expanded sample size and prolonged period of follow-up, so as to elaborate the defects and long-term effects of the proposed treatment scheme comprehensively.

## CONCLUSION

In conclusion, periodontal initial therapy combined with orthodontic treatment has a significant effect on anterior teeth displacement caused by periodontal disease, which deserves promotion clinically.

### Authors’ Contributions:

**YL** and **NG:** Carried out the studies, participated in collecting data, drafted the manuscript, are responsible and accountable for the accuracy and integrity of the work.

**GL:** Performed the statistical analysis and participated in its design.

**XS:** Participated in acquisition, analysis, or interpretation of data and draft the manuscript. All authors read and approved the final manuscript.
